# One-Step Fabrication of Microfluidic Channels in Polydimethylsiloxane: Influence of Laser Power on Channel Formation

**DOI:** 10.3390/mi16030282

**Published:** 2025-02-28

**Authors:** Seong-Yeop Kim, Han-Byeol Son, Hyo-Ryoung Lim

**Affiliations:** 1 Department of Chemical Engineering, College of Engineering, Pukyong National University, Busan 48513, Republic of Korea; ksy3995@naver.com; 2 Major of Human Bioconvergence, Division of Smart Healthcare, College of Information Technology and Convergence, Pukyong National University, Busan 48513, Republic of Korea; bl990413@gmail.com

**Keywords:** microfluidic channels, picosecond laser processing, PDMS fabrication, capillary-driven fluid transport, wearable biochemical devices

## Abstract

Recent advancements in microfluidic technologies have revolutionized their applications, particularly in drug monitoring, continuous biochemical analysis, and real-time physiological assessments. However, the fabrication of microfluidic devices with precise flow control remains constrained by either cost-prohibitive photolithography processes or limited-precision 3D printing techniques. In this study, we propose a one-step fabrication method employing picosecond laser processing to directly create microfluidic channels in (PDMS). This method achieves micron-scale channel precision while significantly simplifying the fabrication process and reducing costs. This approach eliminates the need for additional encapsulation steps, further reducing contamination risks and improving production scalability. These findings highlight the potential of this fabrication method to advance next-generation wearable biochemical devices and personalized healthcare technologies.

## 1. Introduction

Over the past few decades, microfluidics has evolved from simple fluid control systems to sophisticated platforms for advanced biochemical analysis [1,2,3]. Its high surface area-to-volume ratio enables predictable flow dynamics within microchannels, making it ideal for precise fluid manipulation and efficient sample handling [4]. These attributes have driven significant progress in biochemical and biomedical applications, spanning lab-on-a-chip platforms, wearable diagnostic devices, and groundbreaking developments in fields such as drug discovery and medical diagnostics [5,6,7].

Among these applications, a particularly promising avenue is the development of non-invasive, microfluidic-based sensors tailored for personalized healthcare. These devices aim to monitor biomolecules in biofluids, such as sweat and saliva, offering a pathway for continuous and convenient health monitoring [8]. Early iterations, such as sweat patch sensors, demonstrated the feasibility of real-time tracking but were often limited by unstable sample acquisition and contamination risks [9]. To address these limitations, modern microfluidic systems have introduced innovative mechanisms, such as leveraging pressure differences and capillary forces, to enhance sample acquisition and transport. These strategies facilitate reliable biofluid collection at the microchannel inlet and ensure consistent delivery to the sensing region, thereby reducing handling errors and contamination risks while supporting the development of robust, high-performance devices [10].

Despite these advancements, significant challenges persist in the practical fabrication of microfluidic systems. Polydimethylsiloxane (PDMS) remains the material of choice due to its flexibility, optical transparency, and durability [11,12,13]. Soft lithography, the standard method for creating microchannels in PDMS, offers high resolution but is time-intensive and expensive, limiting its scalability [14]. Additionally, plasma bonding, commonly used for sealing PDMS layers, often results in mechanical weaknesses, fluid leakage, and ruptures under pressure [15]. Emerging techniques like 3D printing, while cost-effective, struggle to maintain surface smoothness and dimensional precision, further complicating the manufacturing process.

To overcome the limitations of traditional microfluidic fabrication methods, this study presents a novel, one-step approach using picosecond laser processing to directly create microfluidic channels in stacked PDMS layers. This innovative method eliminates the need for costly and time-consuming encapsulation processes, making the fabrication process more simple and cost-effective. By utilizing the multi-photon absorption of picosecond laser pulses, the method enables micron-scale channel fabrication while demonstrating consistent position along the y-axis. This approach not only simplifies the fabrication process but also provides a scalable solution, positioning it as a promising method for next-generation wearable and diagnostic microfluidic devices, which could have significant implications for personalized healthcare and biochemical applications.

## 2. Materials and Methods

### 2.1. Sample Preparation

Polydimethylsiloxane (Sylgard 184, Dow Corning Inc., Midland, TX, USA) was utilized in the experiments. The PDMS base and curing agent were thoroughly mixed at a weight ratio of 20:1. To enhance laser energy absorption, black pigment (Slic Pig™; Smooth-On Inc., Macungie, PA, USA) was incorporated at a concentration of 2.5 wt%. The mixture was degassed under a vacuum to eliminate air bubbles, thereby minimizing laser beam scattering caused by voids within the PDMS matrix. The degassed PDMS was cast into Petri dishes and cured at room temperature for 24 h. Subsequently, a transparent PDMS layer was cast over the opaque PDMS layer and cured under the same conditions for an additional 24 h. Disposable syringes were used for casting the PDMS layers, with the mixture weight adjusted to achieve the desired thickness. Fabricated samples were cut into squares to minimize variations caused by the meniscus effect near the Petri dish wall.

### 2.2. Channel Fabrication

Laser processing was performed using a commercial 532 nm Nd:YAG picosecond laser (µ-Lab, Kortherm Science Inc., Incheon, Republic of Korea) operating in TEM_00_ mode with a Gaussian beam profile. We used an F-theta lens with a focal length of 240 mm and a numerical aperture (NA) of 0.03. Key parameters, including the Rayleigh length, focal spot size, and pulse duration, are summarized in Table 1. A Galvanometer scanner facilitated precise beam control, while the sample stage allowed accurate positioning along the x, y, and z axes to compensate for variations in sample thickness. The laser power exhibited a stability variation of ±0.3 W with a standard deviation of 0.12. Microfluidic channel designs were created using AutoCAD 2024 and RAYGUIDE software, with patterns saved in DXF format and converted into commands for laser parameter adjustments. The laser power ranged from 1.2 to 12 W, the scan speed varied between 3 and 50 mm/s, and the repetition rates were set between 300 and 900 kHz, with systematic optimization ensuring precise channel fabrication. Following laser processing, the applied power and laser fluence, defined as the energy density of light intensity, were measured using a power meter.

**Figure 1:**
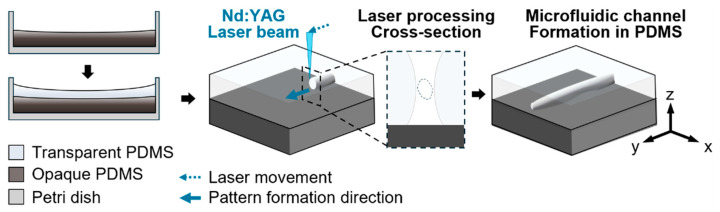
Schematic illustration of the fabrication process for microfluidic channels in multilayer PDMS. The process involves casting a transparent PDMS layer over an opaque PDMS substrate in a Petri dish, followed by picosecond Nd:YAG laser processing. The laser beam is focused on the transparent PDMS layer, forming embedded microchannels.

A conceptual illustration outlines the sample preparation and laser processing workflow for microfluidic channel fabrication in multilayer PDMS (Figure 1). The process begins with the drop-casting of transparent and opaque PDMS layers within a Petri dish, followed by laser irradiation using a 532 nm Nd:YAG laser. The laser beam is focused on the surface of the transparent PDMS, propagating into the material and forming embedded microchannels. This channel formation, along with a detailed analysis of channel morphology, is further explored in Section 3.2 and Section 3.3.

### 2.3. Characterization

The samples were characterized using scanning electron microscopy (SEM; MIRA 4, Tescan Inc., Warrendale, PA, USA) to evaluate interlayer bonding and identify potential fluid leakage at the PDMS interface. Prior to SEM analysis, a thin layer of platinum was sputtered onto the samples to enhance imaging resolution. Cross-sectional views of the embedded channels were examined using optical microscopy (HNM001, GasWorld Inc., Seoul, Republic of Korea) to assess channel connectivity.

### 2.4. Capillary-Driven Flow Test

The fluid transport capabilities of the fabricated channels were evaluated through a capillary-driven flow test. A dyed water solution was introduced into vertically positioned channels, and optical microscopy was used to capture images after the solution traversed the channel.

## 3. Results

The channel fabrication was performed using stacked PDMS samples, where each layer serves a distinct purpose. The transparent PDMS allows laser transmission and absorption when the channel formation position is varied based on laser parameters. The black part has two key functions: (i) acting as a protective barrier to prevent laser exposure to the equipment and (ii) enhancing visibility by providing contrast, making channel observation easier. While these were the initially intended functions of the bottom layer, we further utilized them to extend the experiments by facilitating the expansion of channel diameters by terminating the laser beam on the black PDMS layer.

### 3.1. Channel Fabrication

The channel processing method effectively produced microchannels within the transparent PDMS layer. Cross-sectional and surface analysis using optical microscopy and SEM were conducted to examine the morphology of the fabricated channels. These channels, with widths ranging from 10 to 150 µm depending on laser fluence, were established under optimized conditions, as summarized in Table 2. Additionally, the pulse duration (12 ps) and pulse energy range (3.8 µJ to 32.9 µJ) have been explicitly included, ensuring a more complete description of the operating conditions. The optimized conditions were chosen by varying the laser power while maintaining a constant repetition rate and scanning speed. At a repetition rate of 300 kHz and a scanning speed of 50 mm/s, we achieved comparatively consistent laser patterning, particularly in terms of pattern position uniformity. The laser spot overlap (*L.S.O*) was calculated as a function of the scanning speed to ensure proper overlap of the laser spots:

(1) $$L.S.O=1-(\frac{v}{f\cdot d})\times 100$$

where f is the pulse repetition rate, v is the scanning speed, and *d* is the beam spot diameter.

Microchannels were fabricated exclusively within the transparent PDMS layer using fixed laser settings: Level 2, 6 W power, 300 kHz repetition rate, and 50 mm/s scanning speed, corresponding to a measured laser fluence of 2.9 J/cm^2^.

### 3.2. Channel Formation and Influence of Laser Power

**Figure 2:**
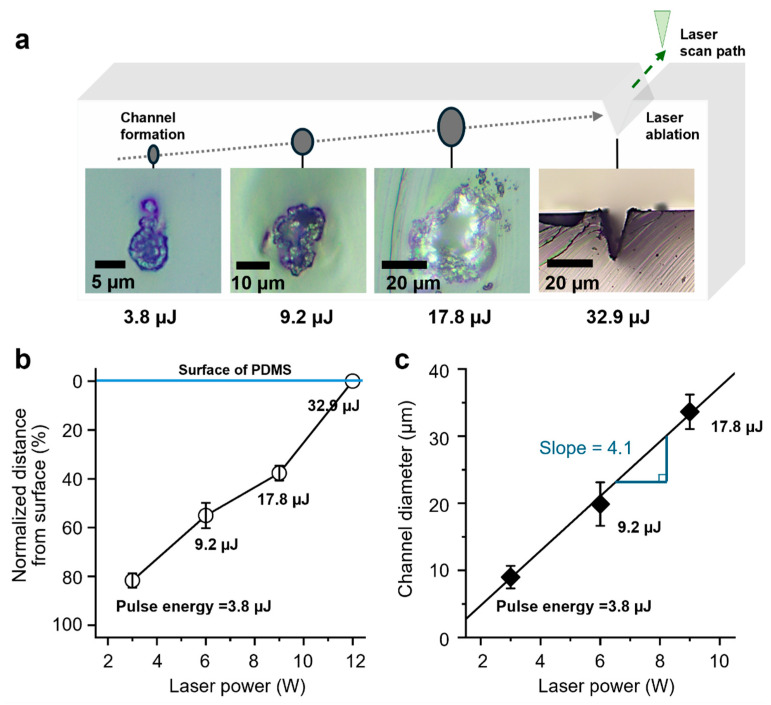
Influence of laser power and channel formation. (**a**) Cross-sectional images of fabricated channels showing variations in pulse energy from 3.8 µJ to 32.9 µJ. The blue arrow indicates laser scan path along the y-axis. The green dashed arrows indicate the movement direction, while the gray dashed arrows represent the positional changes of the pores as laser power increases. (**b**) Normalized fabricated channel position relative to the PDMS surface shifts toward the surface as laser power increases. (**c**) The relationship between laser power and channel diameter exhibits a linear increase, with a slope of 4.1.

The effects of laser power, repetition rate, and scanning speed on channel geometry and placement were systematically investigated. Figure 2 provides optical images of the fabricated microchannels, illustrating the cross-sectional views. Figure 2a demonstrates the influence of laser power on channel morphology and geometry. The channel fabricated at 3 W (1.2 J/cm^2^, 3.8 μJ) shows smooth channel boundaries with minimal surface ablation, highlighting the effectiveness of low-fluence processing. Channels fabricated at 6 W (2.9 J/cm^2^, 9.2 μJ) exhibit a wider channel diameter and early signs of bubble formation due to increased energy absorption. Channels fabricated at 9 W (5.6 J/cm^2^, 17.8 μJ) display notable irregularities, with localized thermal effects causing deformation in the PDMS matrix.

The channels fabricated at 12 W (10.5 J/cm^2^, 32.9 μJ) exhibit significant surface ablation with an aspect ratio of 0.8:1. The green arrow in Figure 2a indicates the y-axis laser scanning trajectory, while the laser beam propagates along the z-axis. The cross-sectional image clearly illustrates highly irregular and uneven geometries that compromise structural uniformity. Additionally, the position of the laser beam waist is approximately (0.2 mm) below the PDMS surface, marking the focal region where energy deposition is maximized, leading to excessive material removal. This indicates that above a specific threshold fluence, laser absorption becomes dominant at the surface, resulting in ablation rather than transmission. Residual material, observed around the channel edges, ranged from 1 µm to 7 µm depending on the applied fluence. These results highlight the importance of optimizing laser power and fluence to achieve uniform channel geometries while minimizing thermal degradation. The aspect ratio was measured through cross-sectional analysis along the y-axis, following the laser trajectory, at 30 points for each mode (Levels 1 to 3). The measured aspect ratios were 0.55:1, 0.74:1, and 0.72:1, respectively, with standard deviations ranging from 0.11 to 0.14, demonstrating elliptical channel morphologies.

Figure 2b,c summarizes the relationship between laser fluence and channel geometry, highlighting the proportional increase in channel diameter with rising laser power until the onset of surface damage. The influence of laser power on microchannel geometry and position was systematically evaluated to identify optimal fabrication conditions.

Regarding the channel position, as laser fluence increases, the depth at which channels form becomes shallower, progressively shifting closer to the PDMS surface. This phenomenon is attributed to enhanced energy absorption at higher fluence levels, which results in more intense localized heating and ablation near the surface. Also, increasing laser power shifted the laser focusing point, leading to deviations in channel placement within the PDMS. In this experiment, the effects of pico-laser irradiation on PDMS were observed at different power levels. At higher power (10 W), the energy is primarily absorbed at the surface, resulting in local heating and the formation of cracks. In contrast, at lower power, the laser energy penetrates deeper into the material, causing energy absorption at greater depths, which leads to the formation of pores. At lower pulse energy, such as 3.8 µJ, the laser energy penetrates deeper into the PDMS matrix, creating channels farther from the surface. However, at pulse energy above 17.8 µJ, the laser energy density exceeds the material’s damage threshold, causing significant surface ablation and compromising the uniformity of channel placement. This is evident in the irregular and less predictable positioning of channels formed at the highest fluence values, which can be detrimental to the functionality and scalability of microfluidic devices.

As for the channel diameter control, a proportional relationship between laser fluence and channel diameter is observed up to a critical threshold. At lower pulse energy, such as 3.8 µJ and 9.2 µJ, the channels maintain relatively small diameters due to the limited energy delivered per pulse. Increasing the pulse energy to 17.8 µJ expanded the channel diameter significantly, reaching optimal size with surface irregularities, such as irregular residuals and bubble formation around the channels. Beyond this fluence, at 10.5 J/cm^2^, the channels exhibit excessive material removal and irregular geometries caused by thermal damage and localized energy overflow. This transition underscores the importance of optimizing laser parameters to maintain consistent channel dimensions and structural integrity.

**Figure 3:**
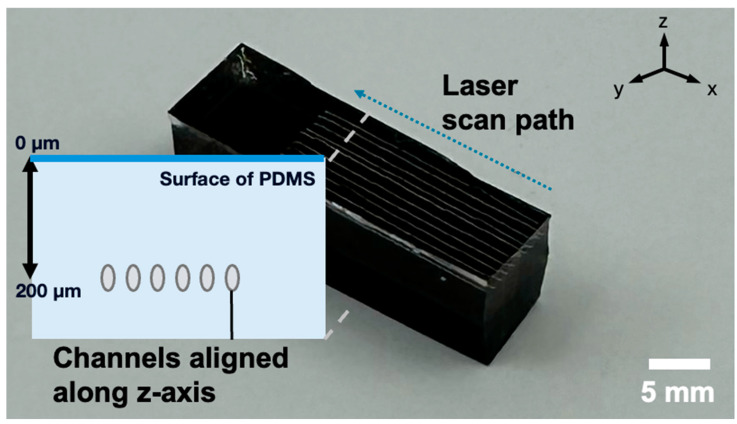
Optical images showing the surface and cross-sectional views of microchannels embedded in transparent PDMS with fixed laser exposure mode: 2.9 J/cm^2^ of laser fluence, a 300 kHz repetition rate, and a 50 mm/s scanning speed.

After investigating the influence of the laser power and channel formation, we control the consistency along the z-axis and the laser scan through the y-axis. The channels formed parallel to the laser spot trajectory (y-axis) (Figure 3) and were embedded approximately 2 mm below the PDMS surface with a spatial variance of ±8%. These results demonstrate that uniform microchannels can be fabricated in polymers by precisely adjusting laser power, repetition rate, and scanning speed, which govern energy deposition and focal depth within the PDMS (z-axis control) (Figure 3). Proper parameter optimization ensures alignment and consistent positioning of the patterned microchannels. However, external factors, such as intrinsic properties of the polymer and surface contaminants like dust, can adversely affect channel connectivity. For instance, the presence of dust particles disrupted the fabrication process, leading to misaligned or incomplete channels. This highlights the importance of maintaining a clean sample surface to ensure reliable channel formation.

### 3.3. Channel Formation at PDMS-PDMS Interface

**Figure 4:**
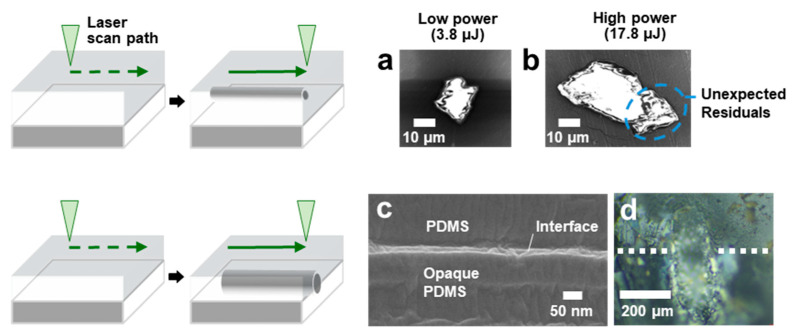
Process of channel formation experiments for improving channel diameter. (**a**) Fabricated microchannels at the PDMS interface using 4 W laser power, a repetition rate of 300 kHz, and scanning speed of 50 mm/s. (**b**) Laser power was increased to 9 W while other parameters remained unchanged. Residuals near the channels indicate the locally concentrated modification. (**c**) SEM analysis of the cross-sectional interface between two PDMS layers. (**d**) Fabricated microchannels at PDMS interface using 7.5 W laser power, a repetition rate of 350 kHz, and scanning speed of 10 mm/s. The images show an increase in channel size. The white dashed lines indicate the interface in (**c**).

Cross-sectional analysis illustrates the irregular morphologies of fabricated channels, showing uncontrolled residuals near the channel at higher pulse energy. The electron beam was set to 5 keV to minimize polymer damage, and an in-beam secondary electron detector was used. Figure 4a,b compares the channels fabricated under isotropic energy transfer and localized thermal effects using 4 W and 9 W laser power, a repetition rate of 300 kHz, and a scanning speed of 50 mm/s. Figure 4b shows irregular morphologies, while the localized heat-affected PDMS matrix exhibited a relatively skewed heat-affected zone (HAZ) at the bottom-right corner.

An increase in laser power leads to a larger channel diameter; however, the limitation in diameter remains due to the shift in channel position, as excess energy causes surface ablation. In addition, uncontrolled residuals become more pronounced as the laser power increases, further limiting the channel diameter scalability. To address this limitation, we directed laser beam propagation toward the PDMS–PDMS interface, where the opaque PDMS enhances the laser absorption due to its high absorptivity. UV-vis spectroscopy measurement shows that the transmittance of opaque PDMS is nearly zero, indicating intensive light energy absorption at a wavelength of 532 nm.

Figure 4c shows a representative SEM image of the interface between the transparent and opaque PDMS layers, illustrating the clean and narrow apertures that effectively prevent fluid leakage. In contrast, when the layers are fabricated separately and bonded using oxygen plasma by grafting hydroxyl groups onto the surface to induce siloxane bonding, the bonding force proved insufficient. This resulted in visible gaps at the interface, leading to easy detachment under light contact. Additionally, the sample preparation process for thick PDMS, typically several millimeters thick, is not adequate for the oxygen plasma bonding process.

Laser parameters were optimized again to control the channel formation position, with scanning speed being the most critical factor. Variations in scanning speed change the refractive index in a transparent medium accordingly [16]. For the channel formation at the PDMS interface, the optimized laser parameters were set as follows: a laser power of 7.5 W, a repetition rate of 350 kHz, and a scanning speed of 10 mm/s. The transparent layer thickness was also optimized to 4.1 mm. By optimizing laser parameters and adjusting the thickness of the transparent PDMS layer, we achieved significant expansion of the channel dimensions, with heights reaching 420 µm and widths up to 130 µm (Figure 4d). The measured fluence was 3.8 J/cm^2^ and the pulse energy was 11.8 µJ. Optical images confirm that the channels were fabricated at the interface between the transparent and opaque PDMS layers, with the opaque layer effectively absorbing the transmitted laser energy to facilitate precise channel formation. This absorption may facilitate channel formation and contribute to structural uniformity.

### 3.4. Mechanism of Channel Formation in PDMS

**Figure 5:**
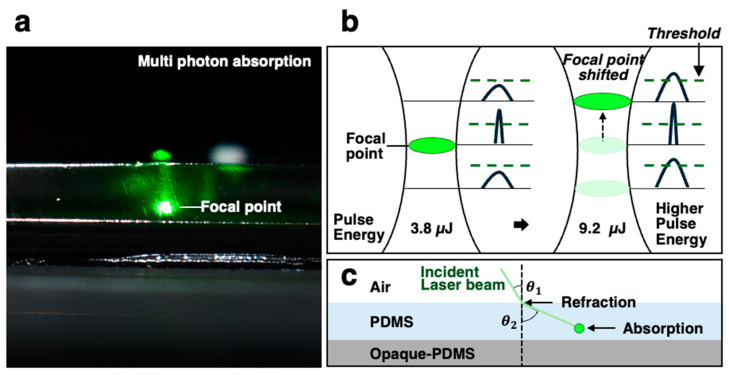
Mechanism of laser processing within PDMS. (**a**) The focal point in transparent PDMS represents the region of multi-photon absorption. (**b**) Schematic illustration showing how increased pulse energy shifts the focal point as the laser fluence exceeds the threshold. (**c**) Schematic depiction of laser beam refraction at the air–PDMS interface and absorption at the focal point.

Multi-photon absorption was observed and recorded using a CCD camera, showing a region of concentrated light in Figure 5a. The laser beam transmitted the PDMS and was simultaneously refracted according to its linear refractive index. The exact refractive index was not measured in this study; however, we estimate the refraction based on the reported value of 1.4348 for transparent PDMS at a 532 nm wavelength [17].

In Figure 5, we have explicitly illustrated (i) the beam propagation path and energy deposition regions within the transparent PDMS layer; (ii) the multi-photon absorption zone, distinguishing it from other energy dissipation areas; and (iii) the influence of laser peak power on channel depth control and how absorption shifts accordingly.

As the laser beam propagates through a transparent medium, multi-photon absorption is initiated when the pulse energy exceeds the threshold of PDMS. We interpret the focal point as being determined by the relationship between pulse energy and this threshold, which is consistent with our results (Figure 5b). The decrease in focal point with the increase in laser power can be attributed to the pulse energy exceeding the threshold earlier, causing the focal points to shift accordingly (Figure 5b).

The data reveal that the optimal conditions for microchannel fabrication occur at fluence levels below 5.7 J/cm^2^. Within this range, channels maintain their consistent positioning, minimizing the risk of surface ablation and deformation. In contrast, fluences exceeding this threshold introduce significant HAZ and thermal damage, which disrupt the precision of the microchannel fabrication process.

The geometry distortion observed in the focal point results from the extension of the optical path length, and as the laser propagates through the air–PDMS interface, refraction occurs due to the refractive index contrast, altering the focal point [18,19]. As the beam continues within the transparent PDMS, this shift in focal position persists, as illustrated in Figure 5c.

Additionally, the consideration of the laser fluence threshold involves understanding how the beam interacts with the PDMS surface: (i) At low fluence, the laser beam propagates through PDMS with an extended focal length, leading to deeper energy distribution. (ii) At high fluence, absorption at the PDMS surface increases and laser ablation occurs due to the threshold-dependent variation in absorption and transmittance [20].

The focal shift inside PDMS can be estimated using an analytical expression derived from the refraction-induced optical path extension and beam propagation characteristics [20]:

(2) $$∆f=l\cdot (1-\frac{f}{\sqrt{n^{2}f^{2}+(n^{2}-1)R_{0}^{2}}})$$

where *f* is the focal length, *n* is the refractive index, and *R*_0_ is the beam diameter at the lens. Based on our system parameters, the calculated focal shifts for levels 1, 2, and 3 are approximately 1.369 mm, 0.879 mm, and 0.197 mm, respectively.

### 3.5. Fabricated Channel Connectivity Test

**Figure 6:**
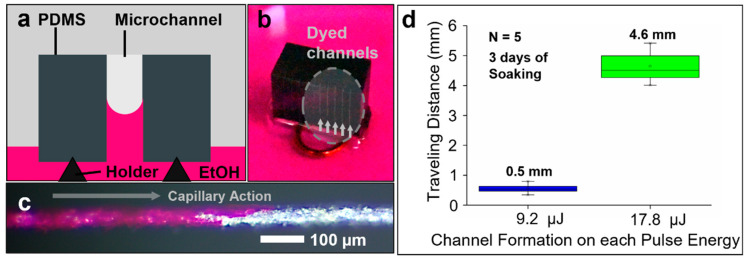
(**a**) Schematic of the capillary-driven flow test setup. (**b**) Optical image of the experiment setup. (**c**) Top-view showing the fluid transverse within the fabricated microchannels. (**d**) Traveling distance of test fluid along the channels. Channel fabrication follows the parameters described in previous results for levels 2 and 3.

The fluid transport performance of the optimized microchannels was evaluated through a capillary-driven flow test. A dyed water solution was introduced into vertically oriented channels, with capillary forces facilitating fluid movement without external pumps. The experimental setup and results are depicted in Figure 6, which demonstrate the fabrication of connected microchannels, allowing for fluid transport without evidence of clogging caused by the laser process.

Channels fabricated under optimized parameters demonstrated successful fluid transport, attributed to their connected channels. The top-view and cross-sectional images in Figure 6b,c illustrate dyed fluid driven into the vertically arrayed microchannel by capillary action. The capillary-driven flow test demonstrated that fluid movement was initiated passively due to capillary forces without the application of external pressure. Additionally, we conducted repeated capillary tests on five samples (level 2), and after 3 days, the test fluid traveled an average distance of 0.5 mm ± 0.15 mm (Figure 6d). At higher pulse energy (level 3), the dyed length increased to 4.6 mm ± 0.51 mm. However, under the tested conditions, passive capillary action alone was insufficient to fully transport the fluid through the entire channel. Instead, the flow stabilized at an equilibrium point, beyond which further movement did not occur within the observation timeframe. This behavior suggests that the extent of fluid transport is influenced by factors such as channel geometry, surface roughness, and wettability. While the fabricated channels facilitate passive fluid transport, additional external forces may be required for complete channel filling in certain applications.

## 4. Discussion

This study demonstrates that picosecond laser processing reliably produces microchannels with consistent geometries under optimized laser fluence conditions. Capillary-driven flow tests confirm the capability of these channels to efficiently transport fluids passively, without external pumps. Optimizing laser parameters revealed a proportional relationship between laser fluence and channel diameter, highlighting the precision and controllability of this fabrication technique. Here, we propose plausible mechanisms of laser–PDMS interactions, focusing on photon-electron dynamics. PDMS, an amorphous material in its uncured state, transmits visible light; however, after curing, its cross-linked structure absorbs light variably, which can hinder consistent laser–material interactions. This study addressed this variability by adjusting laser parameters—power, repetition rate, and scanning speed—to regulate the optical and thermal behavior of the cross-linked PDMS matrix.

Initially, we focused the laser at the air–PDMS interface; however, due to the high transmittance (91% at 532 nm) of PDMS, the beam propagated further into the bulk material. The damping coefficient (γ = 9–12 cm^−1^) suggests a penetration depth of approximately 0.1 cm, which is consistent with our observations [17]. Since our PDMS sample thickness exceeds this depth, the expected beam termination position should remain constant. However, experimental results show a decrease in penetration depth with increasing laser power, strongly suggesting the role of nonlinear absorption effects.

This nonlinear behavior allows us to differentiate between the Kerr effect and multi-photon absorption as the dominant mechanism. The Kerr effect, characterized by self-focusing at higher light intensities, would typically lead to increased penetration depth. However, our results show the opposite trend—penetration depth decreases with increasing laser power, indicating that multi-photon absorption dominates energy deposition. The bandgap of PDMS is significantly larger than the energy of a single 532 nm photon, confirming that single-photon absorption is negligible (Table 3).

Although we did not explicitly measure the nonlinear absorption coefficient (β) in this study, previous reports indicate that PDMS exhibits weak nonlinear absorption unless doped with absorbing species [21]. Further Z-scan measurements would be required to quantify β under our specific experimental conditions. However, based on the reported values, we conclude that multi-photon absorption, rather than the Kerr effect, is the dominant energy deposition mechanism in our study. At higher fluence (≥10.5 J/cm^2^, corresponding to a peak intensity of 8.75 × 10^11^ W/cm^2^), surface ablation becomes the dominant mechanism. Here, photon density exceeds lattice electron density, leading to multi-photon absorption as the primary process for localized energy deposition and material removal. However, experimental verification is needed to confirm the extent of avalanche ionization’s contribution to energy absorption and material ablation in these conditions. Similar processes have been reported in glass and silicon, where selective laser etching and self-focusing enable high-resolution subsurface features. Thermal effects, particularly heat accumulation, play a secondary but critical role in shaping ablation morphology. Scanning electron microscopy (SEM) images reveal localized HAZ, where melting and gas phase transition of the PDMS matrix may occur [22,23,24].

**Table 3:** Summarized optical properties of PDMS at 532 nm wavelength.

Parameters	Symbol	Value	Units	Reference
Linear refractive index	n_1_	1.4348 ± 0.0006	-	[17]
Nonlinear refractive index	n_2_	N.A *	cm^2^/W	[21]
Linear absorption coefficient	α	3.58	cm^−1^	[25]
Nonlinear absorption coefficient **	β	N.A *	cm/GW	[21]
Damping coefficient	γ	9-12	cm^−1^	[17]

* Non-detectable due to thermal artifacts and photodamage in reported experimental range; ** Based on prior studies, PDMS exhibits weak nonlinear absorption unless doped with absorbing species.

Prolonged exposure at high repetition rates exacerbates these thermal effects, contributing to void formation and localized structural irregularities. These observations align with reports of thermal lensing and stress accumulation in glass processing.

The laser processing of PDMS relies on the interplay between multi-photon absorption, avalanche ionization, and thermal effects. Multi-photon absorption initiates the process by driving energy deposition, while avalanche ionization enhances energy distribution in the interior. Nonlinear effects like self-focusing further localize energy, enabling subsurface modifications. These combined mechanisms ensure reproducibility and scalability, paving the way for transparent material internal processing with applications in biomedical devices, lab-on-a-chip systems, and advanced photonics. This one-step fabrication method eliminates the need for traditional encapsulation and bonding steps, significantly simplifying the fabrication workflow and reducing production time and costs. We compared various laser techniques for microfluidic channel fabrication in PDMS (Table 4). This comparison across different processing techniques underscores the unique potential of our work, demonstrating that polymer internal processing remains unexplored. Despite these advantages, challenges remain, including channel irregularities at higher fluence, limited large fluid volume applications, and integration with other microfluidic components. Future research should focus on integrating advanced laser control techniques, such as beam shaping and multi-step etching, to further optimize the fabrication process. The simplicity and effectiveness demonstrated in this study position this method as a promising approach for next-generation microfluidic technologies.

## 5. Conclusions

This study successfully demonstrated a novel one-step fabrication method for microfluidic channels in stacked PDMS layers using picosecond laser processing. The proposed approach eliminates the need for traditional encapsulation and bonding steps, significantly simplifying the fabrication process and reducing production time and costs. The findings highlight the potential of this technique for creating scalable microfluidic devices suitable for various applications, including wearable sensors and biochemical analysis systems. Despite the advantages, challenges such as thermal effects at higher fluences and limited compatibility with large fluid volume applications remain areas for improvement. Future research should focus on optimizing channel designs, expanding the method to diverse materials, and integrating advanced functionalities to further enhance its versatility and scalability. This study lays the groundwork for advancing next-generation microfluidic technologies tailored to personalized healthcare and diagnostic solutions.

## Figures and Tables

**Table 1:** Summarized characteristics of the laser system.

Characteristics	Value	Unit
Wavelength	532	[nm]
Beam shape	Gaussian profile	--
Beam quality (M^2^)	1.11	--
Spot size	12-15	[µm]
Laser mode	TEM_00_	--
Focal length	240	mm
Rayleigh length	192	[µm]
Divergence angle	0.54	[mRad]
Scanner type	Galvanometer	--
Pulse duration	12	[ps]

**Table 2:** Summarized applied laser parameters and measured values.

	Laser Parameters	Unit	Level 1	Level 2	Level 3	Level 4
Applied variables	Power	[W]	4	6	9	12
	Repetition rate	[kHz]	300	300	300	300
	Scanning speed	[mm/s]	50	50	50	50
Measured parameters	Pulse energy	[µJ]	3.8	9.2	17.8	32.9
	Fluence	[J/cm^2^]	1.2	2.9	5.7	10.5
	L.S.O	[%]	99.2	99.2	99.2	99.2

**Table 4:** Summarized microfluidic channel fabrication technique using a laser.

Reference	Laser Source	Pulse Duration	Wavelength [nm]	Materials	Channel Width [µm]	Controlled Axis	Performance
This study	Nd:YAG	Picosecond	532	PDMS	10–150	x-y	Microchannels fabrication within PDMS
[26]	CO_2_	CW	9300	PDMS, PMMA	~80	x-y-z	Uniform microchannels with controlled depth and width
[27]	CO_2_	Nanosecond	9300	PDMS, PMMA, Glass	77–85	x-y-z	Microchannels with controlled width/depth
[28]	Nd:YAG	CW	532	PS/PDMS	4.7–12	x-y-z	3D microchannels in glass
[29]	Nd:YAG	Femtosecond	800	Glass/PDMS	53	x-y	Underwater superpolymphobic microchannels (PCA 155.5°)
[30]	Nd:YVO4	N/A	355	PMMA	20–200	x-y-z	High-quality PDMS mold fabrication with smooth surface finish
[31]	Excimer	Nanosecond	193	PDMS, PGS, APS	10–150	x-y	Precise microchannel fabrication via surface ablation

## Data Availability

Data supporting the findings of this study are available upon reasonable request from the corresponding author.
